# Cochlear Synaptopathy and Noise-Induced Hidden Hearing Loss

**DOI:** 10.1155/2016/6143164

**Published:** 2016-09-21

**Authors:** Lijuan Shi, Ying Chang, Xiaowei Li, Steve Aiken, Lijie Liu, Jian Wang

**Affiliations:** ^1^Department of Physiology, Medical College of Southeast University, 87 Dingjiaoqiao Road, Nanjing 210009, China; ^2^School of Human Communication Disorders, Dalhousie University, 1256 Barrington St. Dalhousie University, Halifax, NS, Canada B3J 1Y6

## Abstract

Recent studies on animal models have shown that noise exposure that does not lead to permanent threshold shift (PTS) can cause considerable damage around the synapses between inner hair cells (IHCs) and type-I afferent auditory nerve fibers (ANFs). Disruption of these synapses not only disables the innervated ANFs but also results in the slow degeneration of spiral ganglion neurons if the synapses are not reestablished. Such a loss of ANFs should result in signal coding deficits, which are exacerbated by the bias of the damage toward synapses connecting low-spontaneous-rate (SR) ANFs, which are known to be vital for signal coding in noisy background. As there is no PTS, these functional deficits cannot be detected using routine audiological evaluations and may be unknown to subjects who have them. Such functional deficits in hearing without changes in sensitivity are generally called “noise-induced hidden hearing loss (NIHHL).” Here, we provide a brief review to address several critical issues related to NIHHL: (1) the mechanism of noise induced synaptic damage, (2) reversibility of the synaptic damage, (3) the functional deficits as the nature of NIHHL in animal studies, (4) evidence of NIHHL in human subjects, and (5) peripheral and central contribution of NIHHL.

## 1. Noise-Induced Hidden Hearing Loss (NIHHL)

Noise-induced hidden hearing loss (NIHHL) refers to any functional impairment seen in subjects with noise exposing history but no permanent threshold shift (PTS). This is different from the conventional definition of noise-induced hearing loss (NIHL), which is based on changes in auditory sensitivity or threshold shift [1]. Therefore, noise exposure recommendations are based on the likelihood that a particular dose of exposure will result in a PTS. Noise exposures that are not expected to cause PTS are thus considered safe.

Physiologically, variations in auditory sensitivity following exposure to noise are largely due to the functional status of outer hair cells (OHCs) in the cochlea, which provide mechanical amplification of soft sounds [2, 3]. Noise exposures that result in only a temporary threshold shift (TTS) have a reversible impact on OHC function, which is manifested by the recovery of otoacoustic emissions (OAE) [4–6] and cochlear microphonics (CM) [7–11]. The functional changes in these measures parallel the recovery of hearing thresholds, as well as the repair of structures such as stereocilia and the tectorial membrane [7, 12]. By contrast, noise exposure at higher levels and/or for longer durations can cause permanent damage to, or even the death of, OHCs and, hence, lead to PTS. Therefore, the OHCs and the structures surrounding them, including the tectorial membrane and the supporting cells, are considered to be the major loci of cochlear damage that result in noise-induced threshold shifts [13, 14].

Although some early reports claimed that reversible noise-induced IHC pathologies were responsible for TTS [15, 16], IHCs are relatively insensitive to noise-induced cell death. However, it has long been recognized that the synapse between IHCs and primary spiral ganglion neurons (SGNs) can be damaged by noise [17–19]. These early studies showed that this manifests mainly as damage to the postsynaptic terminals; however, there is clear evidence from more recent studies that noise induces damage to both pre- and postsynaptic structures. More importantly, disruption of the synapses can be permanent, resulting in degenerative death of SGNs [6]. The finding that damage to ribbon synapses can occur without PTS is significant because of the potential impact of such damage on hearing function. Because the physiological damage is not accompanied by a permanent shift in hearing threshold, it would likely be missed by a standard (i.e., threshold-based) hearing assessment and has thus been referred to as NIHHL.

**Figure 1:**
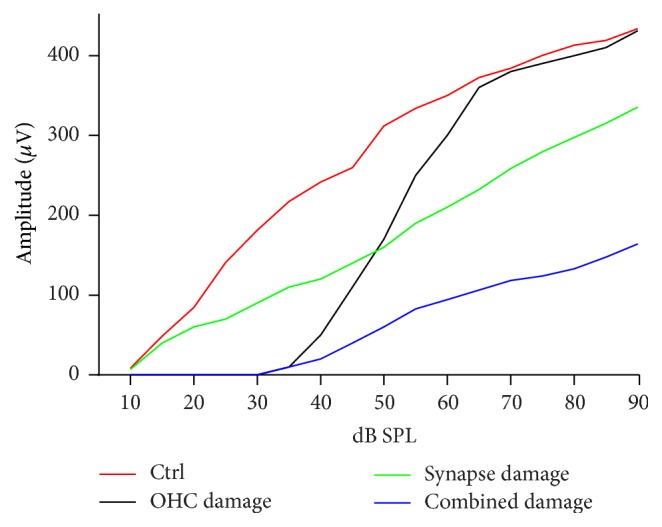
Schematic curves of CAP I/O functions under different conditions. As compared with the control behavior, restricted OHC lesion results in an elevation of CAP threshold, but no reduction of CAP amplitude at high sound levels, while the restricted synapse damage results in the reduction of CAP amplitude largely at high sound levels.

NIHHL first manifests as reduced output of the auditory nerve at high sound levels, without affecting the hearing threshold. This reduction has been found in both animals [6, 20–23] and human subjects with a history of noise exposure but with normal audiograms [24]. Since the thresholds of the auditory nerve remain unchanged, the function relating compound action potentials (CAP) amplitude with sound levels in NIHHL animal is different from that in animals with threshold changes. Schematic curves of CAP input/output functions are presented in Figure 1 for a comparison across normal control and those with different pathologies. Theoretically, if the damage is restricted to OHCs, the major change in CAP input/output (I/O) curve is restricted around threshold and the amplitude reaches the control value at high sound levels. In the case of NIHHL, CAP reduction is mainly at high sound level, with no difference at low sound level, suggesting a suprathreshold deficit. When the damage occurs at both OHCs and the IHC-SGN synapses, the reduction of CAP amplitude is seen across all sound levels.

As NIHHL is initiated at the synapse between the IHCs and SGNs, which silences the auditory nerve fibers (ANFs) that extend from them, the corresponding disorder is categorized as a cochlear neuropathy (i.e., cochlear synaptopathy) [25, 26]. Presumably, the reduction in the amplitude of the auditory nerve response without threshold elevation is due to selective loss of ANFs that have high thresholds, which is supported by single-unit recording studies [20, 27]. Given the important features of those low-spontaneous-rate ANFs in auditory coding, the neuropathy or synaptopathy in hidden hearing loss is not simply a reduction in the number of functional ANFs. Furthermore, the synaptopathy in NIHHL is likely to be related to the synaptic repair after initial damage by noise [20], rather than a simple initial loss. In addition, the functional deficits seen in NIHHL may also involve the contribution from central auditory plasticity [26, 28–32]. In this review, we summarize the available data for noise-induced damage and repair around IHC–SGN synapses and discuss the evidence for the contributions of cochlear malfunction and central plasticity to NIHHL.

## 2. Noise-Induced Damage and Repair around Cochlear Ribbon Synapses

Accumulated evidence has shown that the synapses between IHCs and type-I SGNs are sensitive to noise and the damage to this synapse is likely to be the bases for NIHHL. The synapse is characterized by presynaptic dense bodies termed ribbons [33–35], which are spherical or ellipsoidal in shape, 100–200 nm in diameter [36], and surrounded by synaptic vesicles. The ribbons are built up from RIBEYE protein subunits [37, 38] and anchored to the active zone of the presynaptic membrane via Bassoon proteins [39–41]. The functional role of ribbons has been recognized as tethering and conveying synaptic vesicles to the active zones [42, 43], where the release of neurotransmitters at these synapses is modulated by a specific L-type calcium ion channel (i.e., CaV1.3) [44, 45].

Noise exposure causes damage to both the presynaptic ribbons and postsynaptic nerve terminals of the ribbon synapses [6, 22, 23, 46–48]. The damaged synapses exhibit various degrees of swelling of the terminals, resulting in disruption of the synaptic connections between IHCs and SGNs [20, 46, 48]. Immunohistological staining has revealed similar losses for ribbons and terminals [6, 22, 23, 49]. The mechanism for the damage to the postsynaptic terminal is glutamate-mediated excitotoxicity (reviewed in [46]). However, it is unclear how the presynaptic ribbons are damaged. One possible mechanism of ribbon loss is the loss of cell-cell contact that is required for the maintenance of the pre- and postsynaptic complexes [50–53]. Our electron microscopy evaluations did not reveal any residual presynaptic complexes without ribbon and postsynaptic terminals [20]. Therefore, it is likely that the entire presynaptic structure breaks down when the postsynaptic terminal is damaged.

Another possibility is that ribbon loss results from a breakdown of ribbon building units. A brick assembly model, in which a ribbon is built up from multiple Ribeye subunits, has been proposed for ribbon construction in retina photoreceptor cells [37]. Moreover, the ribbons in retina sensorial cells can be partially broken down by light, but they rapidly reassemble in the dark, probably serving as a mechanism of adaption to bright light [54–58]. In the retina, the ribbon size appears to be a determining factor for the quantity of neurotransmitter released. However, the dynamic disassembling/reassembling process has not been identified in the cochlea, and changes in the ribbon size and the relationship with the release of neurotransmitters have not been investigated in the cochlea. Additionally, disassembly and reassembly, as well as ribbon size, are modulated by Ca^2+^ signaling involving CaV-channels, presynaptic Ca^2+^ levels and storage, and guanylate cyclase-activating protein-2 (GCAP2; see the review by Schmitz [59]). Interestingly, optical stimulation of photoreceptors causes hyperpolarization of the presynaptic membrane and a decrease in [Ca^2+^]_i_, as opposed to depolarization and the large increase in [Ca^2+^]_i_ in IHCs in response to sound. The decrease in [Ca^2+^]_i_ in photoreceptor cells is followed by a conformational change of GCAP2, which results in the disassembly of the ribbons. In the cochlea, it is not known whether there is a GCAP-mediated pathway that controls ribbon size. As the membrane potential of IHCs is depolarized with increasing sound levels, resulting in an influx of Ca^2+^, the role of Ca in ribbon assembly is unlikely to be the same as it is in the retina.

### 2.1. Is the Synaptic Damage Reversible?

There is some debate about whether noise-induced ribbon synapse damage is reversible. The first quantitative study of noise-induced ribbon synapse damage in CBA mice [6] reported that the number of ribbon synapses was reduced to 40% compared with the control 1 day after brief noise exposure that did not lead to PTS. The synapse count recovered to 50% within 1 week, but no further recovery was observed, and this 50% loss of synapses was considered permanent. SGN death observed 2 years after the noise was found to match the 50% permanent loss of synapses [6]. However, a study on guinea pigs carried out by the same research group found a similar loss of ribbon synapses 2 weeks after exposure to noise that did not cause PTS [49], but this study found a much smaller final loss of SGNs. This suggests that some SGNs, which had originally lost their synapses with IHCs, survived and reestablished synapses with IHCs.

Our studies on guinea pigs have revealed a clear recovery in the synapse count following a massive initial loss induced by noise exposure that did not lead to PTS [22, 23]. Although this recovery was not complete, approximately 50% of the initial loss of paired ribbon and postsynaptic density (PSD) puncta in the basal half of the cochleae was seen 1 day after noise, and the loss was recovered to <20% within 1 month. Comparing the aforementioned data from mice and guinea pigs, it appears that there may be some species difference in the ability to regenerate synapses following noise-induced hearing damage. However, a recent study of C57 mice reported that the loss of ribbon synapses induced by non-PTS-inducing noise was largely reversible [60]. This discrepancy in synapse regeneration following noise exposure requires further investigation.

In a recent review, it was argued that the recovery of CtBP2/PSD counts in guinea pig cochleae following noise exposure reported in our studies may be attributable to up/downregulation of the synaptic protein rather than regeneration of synaptic connections [25]. However, there are several lines of evidence for the possibility of synapse repair following noise-induced damage. First, it has been reported that plastic changes occur in the presynaptic component, including the existence of multiple presynaptic ribbons around an active zone [61] and the changes in the size and location of ribbons following noise exposure [22]. Second, the change in the amplitude of the compound action potential (CAP) corresponded to the changes in ribbon/PSD counts: a large initial reduction in CAP amplitude and synapse counts were followed by a significant recovery after the noise exposure [20]. Third, changes in many single-ANF coding activities were not seen at the time that the synapses were damaged but rather manifested later (see Section 3) with the recovery in both the CAP and synapse number, suggesting that those changes occurred in the ANFs that connect IHCs via repaired/reestablished synapses [20]. Further work is required to determine the mechanisms and factors that influence the repair of both pre- and postsynaptic components.

## 3. Cochlear Coding Deficits in Hidden Hearing Loss

Ribbon synapses exhibit spatial differences around IHCs; that is, the synapses at the modiolar side of an IHC have relatively small ribbons but larger postsynaptic terminals, whereas those at the pillar side have relatively large ribbons but smaller terminals [62]. This spatial variation in synapse morphology has been linked to functional variations across ANFs. Liberman et al. reported that ANFs are functionally categorized by their spontaneous rate (SR), which is inversely related to the fiber's threshold and dynamic range [63–65]. It is widely accepted that low-SR ANFs exhibit synapses with IHCs on their modiolar side, whereas high-SR units exhibit synapses on the pillar side (this is based on data obtained using intracellular tracer injections) [66]. The low-SR units are considered critical for hearing in noisy environments due to their larger dynamic range, higher thresholds, and the ability to follow the quick change of the amplitude of acoustic signals. By contrast, high-SR units are responsible for the sensitivity to quiet sounds and are saturated by high-level background noise [26, 63, 64, 67, 68].

In NIHHL, low-SR ANFs appear to be more vulnerable to noise than high-SR units. Selective loss of low-SR ANFs has been found following exposure to noise that did not lead to PTS [27]. Presumably, this selective loss of low-SR units should produce coding deficits, which can be predicted based on the unique features of those units [26]. However, no coding deficits were examined and reported in this study [27]. On the other hand, we reported a time delay in the development of coding deficits by single ANFs in guinea pigs following a similar noise exposure that did not cause PTS [20]; these deficits were attributed to intensity coding and temporal coding as summarized in Sections 3.1 and 3.2.

### 3.1. Intensity Coding Deficits in NIHHL

Intensity coding in the cochlea is defined as the ability of ANFs to encode the sound intensity or the change of sound intensity. This ability is determined primarily by the spike rate (or the change of spike rate) of individual ANF in response to sound intensity change and the number of functional ANFs. Therefore, the intensity coding deficits can be evaluated in both evoked field potential and single-unit recordings. Deficits in intensity coding were first suggested by a reduction in wave I of the auditory brainstem response (ABR) [6, 49], as well as a reduction in the amplitude of the CAP [20], as this is likely due to the loss of functional ANFs following synapse disruption. The fact that the reduction is more significant at higher sound levels has been considered evidence for selective damage to low-SR fibers, which have higher thresholds [25, 26, 69].

The deterioration in intensity coding following no-PTS noise exposure was manifested as a reduction in the driven spike rates (peak, sustained, and total rates) of ANF units that were tested only at one sound level [20]. Such changes are significant only in low-SR ANF units and are seen at a later time rather than immediately following exposure. This time delay in the development of coding deficits suggests that (1) the reduction in driven spike rates occurs in the ANFs to which the synaptic connections to the IHCs are reestablished following the initial disruption and (2) the repaired synapses are functionally abnormal, with less efficient neurotransmitter release.

### 3.2. Temporal Coding Deficits in NIHHL

Temporal processing ability in the cochlea as well as in the whole auditory pathway is defined as the ability to follow the quick change of acoustic signals. In human subjects, the process involves both bottom-up and top-down mechanisms; but in animal models, only bottom-up mechanisms are tested (see reviewed by [67]). Many different tests have been used to detect the bottom-up mechanisms of temporal coding, some of them based on the peristimulatory changes of firing rate showing latency and adaptation. As reviewed above, the major function of presynaptic ribbons in IHCs is to facilitate the synaptic transmission. Therefore, the damage to this synapse likely produces temporal coding deficits in ANFs. Indeed, such deficits were manifested as an increase in response latency of ANFs in animals with NIHHL. This was first demonstrated as a significant delay in CAP peak latency [21] and then further supported by the delayed latency of peak in PSTH (or peak latency) of ANFs in our single-unit study [20]. In another very recent report, such delay was reported in ABR as the marker of cochlear synaptopathy [70]. We also found a reduction in the ratio of peak to sustained rates in animals that were exposed to noise. This ratio is considered an index of the ability of a neuron to encode dynamic signal changes (see review by [68]). Using a paired-click paradigm, we found that the ANF response of noise-exposed animals to the second click recovered more slowly from the masking effect of the first click. These results reveal poorer coding to the transient features of acoustic signals by ANFs, which were examined in previous studies to show the deterioration in of temporal coding in animals with Bassoon mutation [39, 41]. Whereas an increase in peak latency was seen shortly after exposure to noise, changes in the peak rate and the peak/sustained spike ratio, as well as a slower recovery of the spike rate to the second click, were not seen until later, suggesting an association between the deficits and the synapse repair.

Phase locking is a mechanism for the auditory coding of temporal envelopes. A temporal deficit in phase-locking responses has been proposed based on selective loss of low-SR units and the functional features of this group of ANFs [25, 26, 69], but it has not been tested at the single-unit level.

## 4. Association of Coding Deficits with Unhealthy Synaptic Repair 

So far, there appear to be two models for the development of coding deficits in NIHHL. One model suggests that the coding deficit or synaptopathy is simply due to the loss of low-SR ANFs. Since those units have unique functions in signal coding, the loss of those functions is predicted as the consequences. Evidence from our own laboratory suggests another model. That is, the coding deficits are developed as the result of unhealthy synaptic repair after initial disruption. We found that the noise-induced synaptic damage in guinea pigs under NIHHL is largely repairable, leaving only a small amount of synapses not being reestablished. Therefore, the coding deficits or synaptopathy cannot be simply attributed to the loss of SR units. Since the coding deficits are seen at the time when the synapse counts are largely recovered, we believe that the coding deficits likely occur in the repaired synapses (most of them innervating low-SR ANFs). Studies are needed to verify which model is more likely the case in human subjects.

## 5. Central and Peripheral Contributions to NIHHL

Hearing loss impacts auditory perception. It has long been recognized that subjects with normal audiograms may have perceptual difficulties, and this is especially true in the elderly. Age-related hearing loss with threshold elevation is termed peripheral presbycusis, whereas the perceptual difficulties seen in the elderly without threshold shift are usually termed central presbycusis [71]. For example, temporal processing deficits and difficulties of hearing in noisy environments are two major problems experienced by the elderly. These problems were recognized long before the discovery of cochlear damage associated with NIHHL and were considered to be the result of “central auditory processing disorders” [71–75]. It was generally accepted that any perceptual deficits observed without changes to hearing thresholds and cognitive functioning can be attributed to central dysfunctions.

Based on recent progress in functional deficits in cochlear coding, such separation between peripheral and central presbycusis is likely to be incorrect. The so-called central presbycusis may, at least in part, result from disorders in the auditory periphery. The coding deficits related to the loss of low-SR ANFs had been described as a type of auditory neuropathy and/or synaptopathy even before any of the predicted deficits were identified. Data on changes in the SR distributions of ANFs suggest the reestablishment of synapses following an initial disruption that was selective to low-SR units [20]. Although our data revealed abnormalities in some aspects of coding in the auditory nerve, further work is required to investigate coding deficits in NIHHL. Such studies cannot be replaced by speculation based on the selective loss of low-SR fibers; for example, one cannot be certain how the auditory nerve changes its response to amplitude modulation until it is measured at the single-unit level. Two possibilities must be considered: (1) the surviving ANFs may change their function and (2) the initially lost low-SR fibers may be repaired but with changed function.

It should be noted that there is now a tendency in the literature to consider NIHHL to be a purely peripheral issue, a result of the overcorrection of the “central presbycusis.” However, despite the strong evidence for a peripheral contribution, the central contribution to the problems seen in NIHHL should not be neglected. In other words, it may be more constructive to assume that there are both peripheral and central contributions to NIHHL. It is well known that hearing loss (with elevated threshold) can induce central changes, which can result in deteriorations in signal processing. Studies aiming to distinguish the role of central plasticity from that of ribbon synapse damage are rare. One such report found that an increase in central gain was responsible for tinnitus in human subjects with typical damage seen in NIHHL (i.e., reduced auditory nerve input to the brain (measured as a smaller ABR wave I)) but normal hearing threshold [24]. In an earlier study in rats, tinnitus was found 6 months after exposure to noise that caused minimal loss of hair cells and PTS but significant loss of ANFs [76].

One of the central impacts of hearing loss due to damage to peripheral auditory organ is imbalance between excitation and inhibition, resulting in hyperactivity and/or hyperresponsiveness in the central auditory system (see reviews in [77–80]). The types of hearing loss producing such central enhancement include cochlear ablation, drug- and noise-induced damage. While direct effect of drugs and noise on central neurons needs to be differentiated, a similarity across those hearing loss models is the reduction of cochlea output to the auditory brain, which may be the main initial factor causing the imbalance between excitation and inhibition. In this sense, central plasticity should also be seen in subjects with NIHHL. While most of studies in central plasticity using NIHL model correlated the central enhancement with the amount of threshold shifts [29, 81–85], at least one study has reported central enhancement in mice exposed briefly to noise at a moderate level that did not cause PTS, presumably producing only NIHHL [30]. Unfortunately, the reduction in auditory input from the cochlea was not quantified in this study. Taken together, available data suggest that cochlear damage, with or without threshold elevation, can lead to central plasticity by reducing input from the auditory nerve. Further work is required to establish the central contribution to coding/perception difficulties in NIHHL, and previous studies on central processing disorders in subjects with NIHL should be reevaluated to differentiate the central contributions from the peripheral ones.

**Figure 2:**
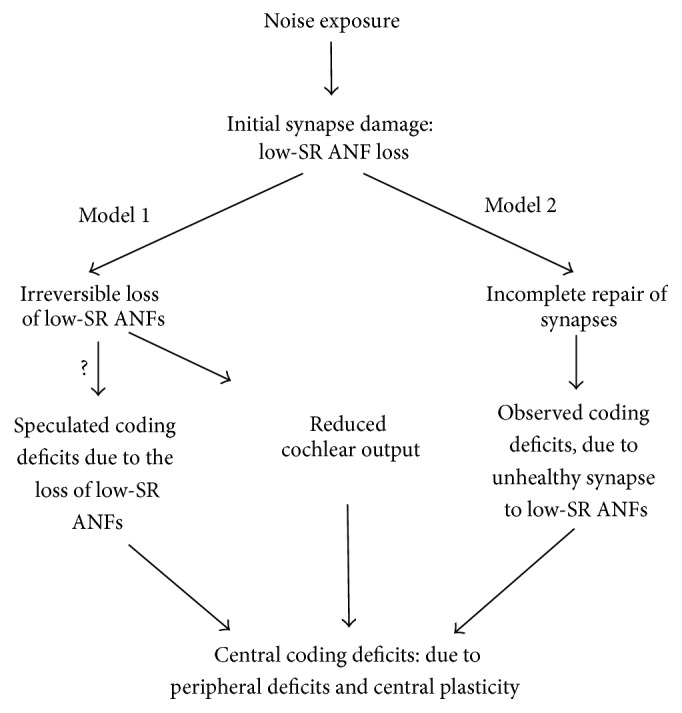
Diagram for the hypothesis of coding deficits in NIHHL.

In a brief summary, we use Figure 2 to summarize the available data for the mechanisms of perception difficulty experienced by subjects with history of noise exposure but normal or near normal thresholds. In this schematic diagram, we include the two potential models of noise-induced synaptopathy in cochleae. In model 1, the coding deficits are speculated based on the role of low-SR ANFs in signal coding. Further evaluation is needed to validate this model. Both models result in a reduction in the cochlear output to the auditory brain, which in turn will result in plastic reorganization of the brain. Auditory signal processing disorders experienced by subjects with long-term NIHHL should include what are inherited from the coding deficits developed in the auditory peripheral and those associated with the plastic changes of auditory brain.

## 6. Clinical Implications and Future Directions

### 6.1. Evidence of NIHHL in Human Subjects

Although more studies on the impact of noise on human hearing showing no changes in auditory sensitivity are required, evidence suggesting the occurrence of NIHHL in human subjects is being accumulated. This is supported by thorough research on the signal perception deficits experienced by subjects with a history of noise exposure but normal thresholds [26]. Since the deficits are demonstrated at suprathreshold levels, it is clear that normal hearing thresholds do not guarantee normal hearing functions, especially in subjects with history of noise exposure [24, 86]. The second line of evidence is the reduction in the output of the auditory nerve in subjects with a history of exposure to noise. This manifests as a reduction in wave I in the ABR at suprathreshold levels [24]. Interestingly, the combination of a reduction in wave I and an increase in wave V/I ratio may be considered evidence of increased central gain and is likely responsible for the generation of tinnitus in hidden hearing loss [32, 87, 88]. The third line of evidence comes from the age-related SGN degeneration seen in the examination of human temporal bones [89]. Unfortunately, there is, as yet, no clear human evidence that degeneration of SGNs is expedited by exposure to noise that does not cause threshold elevation.

### 6.2. Significance of NIHHL

The clinical implications of NIHHL are manifested by the fact that noise exposure causing NIHHL occurs frequently in daily life and impacts much more general population [90]. Such noise exposure has been generally considered to be safe according to current safety standards for exposure to noise. The evidence from the studies reviewed here indicates that the resulting damage to the ribbon synapses from noise that did not induce PTS can be repaired even though the repair is incomplete. More importantly, the signal coding deficits are developed in association with the synapse repair. Since the damage and repair occur repeatedly, the damage on signal coding can be accumulated during aging and likely contributes to the perceptual difficulties experienced by the elderly [26]. This impact of noise exposure on signal coding is obviously different from the contribution made by the hearing loss defined by threshold shifts.

### 6.3. Future Direction

In future, the coding deficits and related synaptic repair in NIHHL should be further investigated in a laboratory setting. Since the ribbon synapse is the first gating point for temporal processing in auditory pathway, the observed coding deficits suggest a clear peripheral origin for the decline in temporal processing and perceptual difficulties during aging. Whether and how the synaptic damage will impact the central auditory processing need to be investigated in a manner that is clearly differentiated from the impact of hearing threshold shift. Moreover, the coding function of ANFs should be observed over a long period of time following exposure to noise to determine whether the coding deficits are temporary or persistent. We are currently collecting data using electron microscopy, as well as conducting an analysis on the potential changes of the molecular structures of ribbons and PSDs, in an attempt to elucidate the morphological/molecular mechanisms responsible for functional changes of repaired ribbon synapses. It would also be interesting to understand the reasons for the extreme sensitivity of low-SR synapses to noise, as well as elucidate possible methods to prevent damage. Laboratory studies should also aim to explore the mechanisms of synaptic repair in the cochlea, as well as reveal the factors that influence repair in order to promote it.

To translate the knowledge to clinic, investigation is needed to establish good measures for detecting NIHHL in human subjects. Although ABR wave I is useful for evaluating synaptopathy caused by noise that does not induce PTS, its reliability and sensitivity are questionable in human subjects where the ABR amplitude is small, and other methods should be explored. A very recent report suggests the use of ABR latency as the marker of NIHHL [70]. The study tested human subjects with normal hearing thresholds and reported a big variation in the threshold of envelope interaural timing difference, which was negatively correlated with the shift of ABR wave V latency by background noise: the higher the threshold (poorer sensitivity), the smaller the shift. The observation of the latency shift with masking is supported by the fact that the low-SR ANFs have longer latency than high-SR fibers and are resistant to background noise [91, 92]. It is not clear why the study did not report the change in wave V amplitude by masking. Theoretically, the masking should produce greater reduction in wave V amplitude in subjects with selective loss of low-SR units. Moreover, no information about the history of noise exposure was reported and it is not clear whether the poorer performance in temporal cue detection was due to noise-induced synaptopathy or other reasons.

To date, the most promising methods for diagnosing cochlear synaptopathy are related to selective loss of low-SR ANFs, the subcortical steady state responses (SSSR) [93, 94]. Based on the animal studies, this test should be carried out using amplitude-modulated signals at relatively high intensity and a shallow modulation depth [95]. The input intensity of the driving signal should fall within the saturation range of the high-SR fibers. High frequency carrier waves with a high intensity and with shallow amplitude modulation are especially useful for evaluating the function of low-SR fibers. This is supported by modeling the loss of low-SR fibers. To differentiate the SSSR contribution from the auditory nerve from that of central neurons, a higher modulation frequency should be used. The optimal modulation frequency is likely to differ across species [96–98]. A recent mouse study found that the modulation frequency close to 1 kHz was optimum with a high frequency carrier without concern of modulation depth [99]. However, a recent human study reported a successful detection of the low-SR unit loss using off-frequency maskers and a shallow modulation depth [95].
